# Experimental Demonstration of Temperature Sensing with Packaged Glass Bottle Microresonators

**DOI:** 10.3390/s18124321

**Published:** 2018-12-07

**Authors:** Jonas Herter, Valentin Wunderlich, Christian Janeczka, Vanessa Zamora

**Affiliations:** Fraunhofer Institute for Reliability and Microintegration, Gustav-Meyer-Allee 25, 13355 Berlin, Germany; jonas.herter@izm.fraunhofer.de (J.H.); valentin.wunderlich@izm.fraunhofer.de (V.W.); christian.janeczka@izm.fraunhofer.de (C.J.)

**Keywords:** bottle microresonators, whispering gallery modes, temperature sensing, packaging, optical sensors, 3D printing

## Abstract

Whispering gallery mode (WGM) glass bottle microresonators are potential highly sensitive structures for a variety of physical and bio-chemical sensing applications. In this paper, we experimentally demonstrate the practical use of glass bottle resonators as temperature sensors. The basic parameters, such as WGM resonance wavelengths, free spectral ranges, and *Q* factors, have been investigated by coupling light from a tapered fiber to the bottle structure. We show the spectral characteristics of the WGMs by choosing different bottle dimensions and taper diameters. For practical measurements, a robust 3D-printed package that includes the bottle resonator and the tapered fiber has been proposed. The packaged bottle has a central diameter D_c_ = 207 µm and a length L = 300 µm. Temperature sensing experiments were also performed. A linear response of the WGM shifts as a function of the temperature is confirmed. The fitted experimental data indicate a temperature sensitivity of 10.5 pm/K at λ ~ 1550 nm, resulting in a limit of detection of 0.06 K. These values can be compared with values reported for other WGM resonators. Additionally, bottle resonators are made with simple splicing methods and their assembly method can be easily defined due to large coupling tolerances.

## 1. Introduction

Optical microresonators [1] are one category of highly sensitive platforms that have been intensively researched over the last two decades. The strong confinement of optical modes (whispering gallery modes (WGMs)) in dielectric microstructures gives rise to unique characteristics such as high sensitivity, compactness, and low power consumption. Typical WGM resonators include spheres [2], disks [3], toroids [4], and capillaries [5]. They have been mainly demonstrated for bio-chemical sensing and environmental monitoring. However, all types of optical resonators are susceptible to temperature changes due to the thermo-optic and thermal expansion coefficients of their material [6]. These effects originate wavelength shifts of the WGMs. In this context, the influence of the temperature in WGM resonators needs to be known in order to develop calibrated temperature sensors. A temperature sensitivity of 14 pm/K at λ = 1531 nm was reported in Reference [7] using glass sphere resonators while 0.2 nm/K at λ = 775 nm was recently obtained with exotic silica bubble resonators [8]. Such results are also compared with the corresponding sensitivities of the well-known temperature sensors based on fiber Bragg gratings [9], surface plasmon resonance [10], and planar ring resonators [11].

In contrast to typical WGM resonators, bottle resonators possess a singular geometry that allows weak confinement of light along their curvature profile. This permits the observation of a high dense spectrum, which has been exploited for the development of microlasers [12], add-drop filters [13], and quantum electrodynamic cavities [14]. However, this spectral dense characteristic can be an obstacle for the implementation of bottle resonators as sensing structures. Despite this fact, filtering methods of WGMs in glass bottle resonators have been proposed and experimentally explored by accurately placing high-index liquid drops and creating micro-scars on the bottle surface via high-precision focused ion beam milling [15]. The cleaner spectrum shows WGM resonances with quality (Q) factors of 10^7^ for high-index dropped bottles and Q ~ 10^5^ for microstructured bottles. The bottle dimensions were central diameters D_c_ = 218 µm, D_c_ = 265 µm, and length L = 550 µm, respectively. As an alternative method, bottle resonators have been proposed with a nanoscale diameter variation and a length of several hundreds of microns. With this structure, a detailed theoretical analysis was reported [16] where a temperature sensitivity was found to be ~10 pm/K. However, the proposed configuration has not been experimentally demonstrated as a temperature sensor. Recently, optofluidic resonators [17] have been the focus of attention due to their natural fluidic channels, which avoid the use of microfluidic integration. Thus, assembly and packaging approaches need to be investigated for bottle resonators in order to be exploited as temperature (or refractive index) sensors.

In this work, we present first experimental results of a packaged glass bottle resonator to detect small temperature changes. The fabrication of the bottle resonators is carried out using a standard splicing tool. A tapered fiber (taper) is used to observe WGMs of a bottle structure. In the optical characterization, different structural parameters of the bottle and the taper are investigated. To demonstrate temperature changes of WGMs in bottle structures, a compact 3D-printed package concept has been proposed and tested over 16 days. The first temperature measurements of the packaged bottle yield a sensitivity of 10.5 pm/K, being comparable to the theoretical expectation.

## 2. Materials and Methods

### 2.1. Bottle Resonators and Tapers

**Figure 1:**
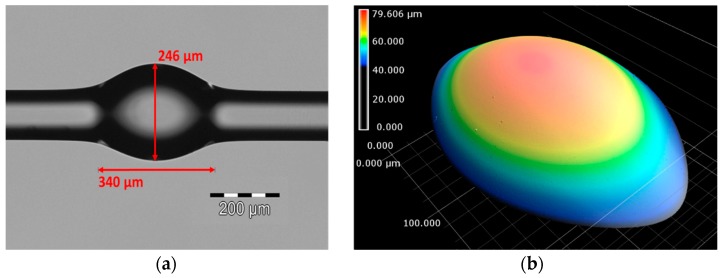
(**a**) Microscope image of the glass bottle resonator (central diameter (D_c_) = 246 µm and length (L) = 340 µm) fabricated with a soften and compress technique; (**b**) the corresponding laser-scanned microscope image of the bottle surface.

Glass bottle resonators are bulges formed by softening and compressing standard single-mode fibers [18]. The proposed soften and compress process was carried out using a commercial fiber splicing tool (Model: FSM100P+) from Fujikura Ltd. In order to create the bulge up to several hundred microns, a continuous piece of fiber was heated by arc discharge and simultaneously compressed. This combined process was repeated several times by re-clamping the fiber, resulting in a bottle resonator with central diameter (D_c_) and bottle length (L). The number of arc discharges used for this work was from 10 to 25 times with an arc duration of one second. Several bottle structures were fabricated with D_c_ ranging from 180 to 250 µm and L between 300 and 400 µm. To produce even smaller bottle resonators, the standard fiber of 125 µm in diameter was replaced by an 80 µm-diameter fiber. The resulting parameters were estimated to be D_c_ = 136 µm and L = 136 µm. All these samples exhibit unique spectral characteristics but only some of them will be briefly discussed in Section 3. Figure 1a illustrates a microscope image of a fabricated bottle structure with estimated parameters of D_c_ = 246 µm and L = 340 µm. The surface of this sample was analyzed via a laser-scanning microscope with resolution of <1 nm. The scanned image in Figure 1b shows a clean and smooth surface. It is well known that ultra-smooth surfaces in fiber-based bottle resonators assure ultra-high Q factors.

In order to evanescently couple light to bottle resonators, adiabatic-profile tapers were produced using a home-made fusion & pull setup and selecting the burner as a heat source [19]. The pendulum motion of the burner and the two pull stages were controlled by a computer. The size of a standard single-mode fiber can then be reduced down to 3 µm. The explored process yielded tapers with total lengths around 28 mm. The corresponding transmission values for those tapers were found to be approximately 94%.

### 2.2. Analysis of Bottle Resonators

The unique geometry of the bottle resonators presents radial confinement but also weak confinement of light in the axial direction. This is mainly caused by the variation of the diameter between two axial caustic points. Here, a better modal confinement can be obtained than, for instance, cylindrical resonators [5]. This phenomenon has a significant impact on the WGM spectrum and optical properties of the bottle structure. Therefore, we have performed an analysis study of the fabricated bottles in order to identify the WGMs and consequently defined the distance between two consecutive WGMs, i.e., free spectral range (FSR). The theoretical study is similar to those in cylinders, but in this case, the radius varies as a function of the axial position R(*z*) and strongly depends on the curvature of the resonator profile [16]. The coupled wave equation obtained from Maxwell equations in cylindrical coordinates splits into two separate radial and axial wave equations by considering the approximation of neglecting the weak *z* dependency. To solve these equations, we have performed simulations in Python and LabVIEW. The latter provides an estimation of the geometrical parameters of the fabricated bottle structure via a distance and contrast contour tool. The central radius (R_c_ = D_c_/2) was determined by setting the region of interest with the distance tool, while the contrast contour along the bulge was used to draw its curvature profile. A fit function of the corresponding profile defines the curvature coefficient (Δk) of the bottle resonator. The parameters, R_c_ and Δk, are used to find the solutions that satisfy the decoupled equations. The radial solutions with order *p* (equal to 1 for the fundamental mode) are based on Airy functions while the axial solutions are defined by Hermite polynomial functions. Such solutions calculated for TE and TM polarization can be denoted by three modal numbers: azimuthal order (*m*), radial order (*p*), and axial order (*q*). Following the procedure in Reference [16], the resonant wavelength solutions are given by:

(1) λm,p,q=2πnb[(Um,pRc)2+(q+12)ΔEm]−12

with

(2) $$\Delta E_{m}=2\frac{U_{m,p}\cdot \Delta k}{R_{c}}$$

and

(3) $$U_{m,p}\approx m\left[1+\frac{\alpha_{p}}{2^{1/3}m^{2/3}}-\frac{n_{\mathrm{out}}}{m{\left(n_{b}^{2}-n_{\mathrm{out}}^{2}\right)}^{1/2}}{\left(\frac{n_{b}}{n_{\mathrm{out}}}\right)}^{\pm 1}+\frac{0.3\alpha_{p}^{2}}{2^{2/3}m^{4/3}}\right]$$

where + and − are chosen for TE- and TM-WGM modes and α*_p_* represents the *p*-th root of the Airy-function. The parameters n_b_ and n_out_ correspond to the refractive indexes of the bottle and the surrounding medium, respectively.

### 2.3. Experimental Setup

**Figure 2:**
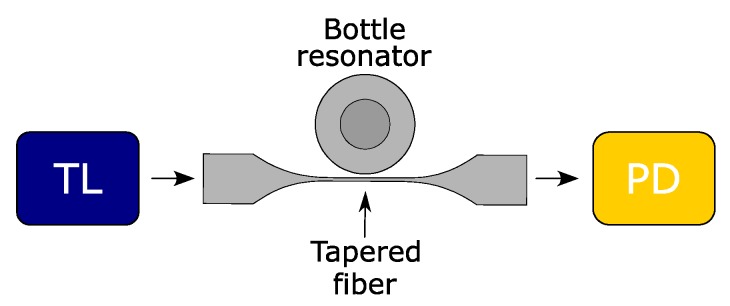
Schematic of the experimental setup. TL: tunable laser, PD: photodiode sensor.

The spectral properties of the bottle resonators were explored using an optical measurement setup. This includes a tunable laser (TL) from Agilent Technologies (Model: 81960A), a low-noise InGaAs photodiode sensor (PD) from Agilent Technologies (Model: 81634B), and optical/mechanical components. The TL source with 100 kHz linewidth and 25 mW maximum power at λ = 1550 nm was used to continuously scan the WGM resonances over a maximum spectral range of 125 nm. The minimal step size was 0.1 pm. The output fiber of the TL was connected to the tapered fiber as shown in Figure 2. Afterwards, a PD collected the transmitted light through the taper. Two micro-positioning stages and charge-coupled device (CCD) cameras were also utilized to tangentially align the bottle resonator with respect to the taper. The taper was in physical contact with the bottle resonator (null gap) for all measurements.

### 2.4. Package

**Figure 3:**
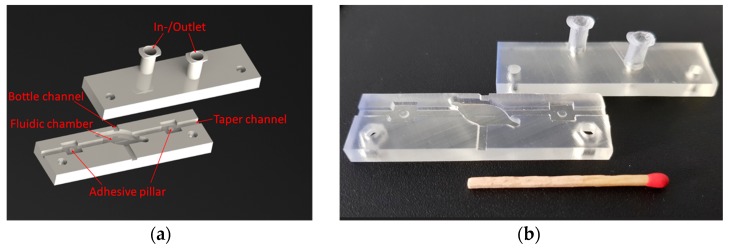
Package for bottle resonators: (**a**) CAD design and (**b**) 3D printed plastic package.

The proposed package for bottle resonators was fabricated using a stereolithography apparatus (SLA) from Formlabs (Model: Form 2” SLA 3D Printer). The laser-based 3D printing technology produces printed plastic pieces from a 3D CAD model by converting liquid photopolymer resin material into solid pieces. One advantage of this process resides in the possibility of rapid prototyping with high accuracy and high surface quality. The package design of a single bottle resonator consists of two parts, base and cover, as illustrated in Figure 3a. The base had a small chamber and two 2.5 mm-wide channels to place perpendicularly both fiber elements (bottle and taper). Additionally, the taper channel had two auxiliary pillars to attach the taper, where a small amount of epoxy was dispensed on the pillar top. In the cover, the inlet and outlet ports were designed as luer-shaped tapers to easily introduce inert gas (or even liquids) into the chamber. The fabricated plastic package in Figure 3b had a small size of 60 × 17 × 10 mm^3^ and can be made with fabrication tolerances of ±100 µm.

## 3. Results and Discussions

### 3.1. Optical Characterization

**Figure 4:**
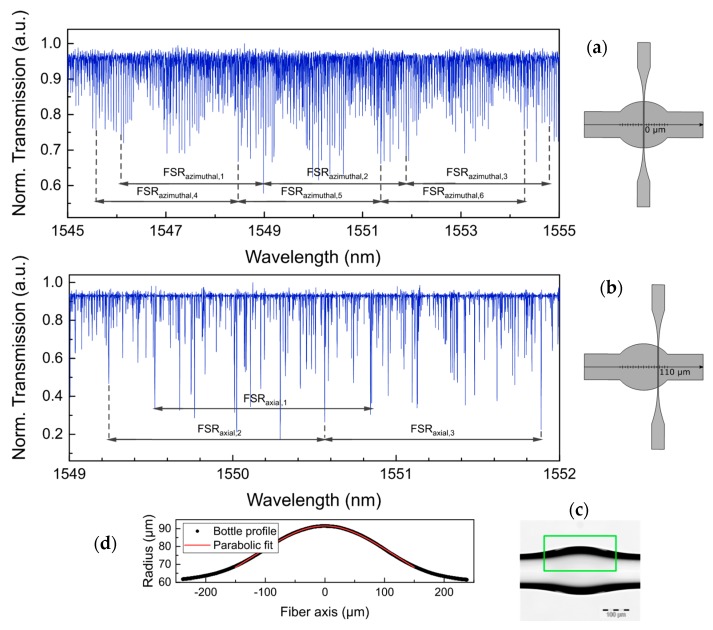
Transmission spectrum of the WGM resonances excited (**a**) in the center of the bottle and (**b**) at a shifted distance of 110 µm. The experimental free spectral ranges (FSRs) are displayed in Table 2; (**c**) microscope image of the interrogated bottle resonator with a taper of 3 µm, with a parabolic profile, D_c_ = 183 µm and L = 362 µm; (**d**) parabolic fit of the bottle profile resulting in a curvature value of Δk = 0.00593 µm^−1^. Step size was 0.1 pm.

The spectral properties of a bottle structure with D_c_ = 183 µm and L = 362 µm have been firstly investigated. WGM resonances were excited with a 3 µm diameter taper located near to the center of the bottle and at a position shifted by ~110 µm from the center to the left. The recorded spectra are shown in Figure 4a,b. A highly dense number of WGMs was observed when the taper was placed in the center of the bottle structure, as shown in Figure 4a. At this position, all WGMs can be excited but only WGM resonances with low *q* and large m dominate the spectrum. They are similar to degenerated WGMs observed in deformed microspheres [18]. When the taper was shifted ~110 µm (Figure 4b), the selective WGMs exhibited large q orders. To identify some WGM resonances with these orders, the theoretical study briefly described in Section 2.2 was implemented. From the microscope image in Figure 4c, the curvature profile of the bottle was determined by fitting a parabolic function, as shown in Figure 4d. The corresponding fit equation is given by:

(4) R(z)=Rc[1+(Δk·z)2]−12=91.5 µm1+(0.00593 µm−1·z)2 from−181 to 181 µm,

where the curvature value was found to be Δk = 0.00593 µm^−1^. The numbers of an individual set of axial orders were approximated by the position of the tapered fiber with respect to the bottle resonator. For this purpose, the following relation for the calculation of the caustic position was used:

(5) zc=[4ΔEm(q+12)]12.

By using Equation (5), two illustrative examples for z = 0 and z = 110 µm were calculated and displayed in Table 1. A TE-WGM resonance of a parabolic-profile bottle (D_c_ = 183 µm and Δk = 0.00593 µm^−1^) at z = 0 was found to be *q* = 0, while this at z = 110 µm turns into a value of *q* = 175. A similar behaviour was observed in the TM-WGM.

In Figure 4a,b, family groups of WGM resonances can be distinguished along the measured wavelength range. The separation of these groups, FSR, has been identified as shown in Table 2. Experimental FSR values (FSR_azimuthal_) for different azimuthal WGM orders were found here to be around 2.9 when the taper was placed in the center. At z = 110 µm, FSRs (FSR_axial_) related to WGMs with large *q* orders were indicated to be about 1.32. The theoretical FSR_azimuthal_ was calculated from two consecutive *m* orders when the *q* order is fixed and *p* = 1. For the corresponding FSR_axial_, this was obtained from two neighboring *q* orders while the *m* order is kept constant and *p* = 1. The theoretical values for both polarizations are displayed in Table 2. These calculations show values of FSR_azimuthal_ ~ 2.9 at z = 0 and FSR_axial_ ~ 1.32 at z = 110 µm. It is easy to see that the FSR_axial_ is always smaller than the FSR_azimuthal_. The theoretical and experimental results exhibit good agreement, however, the high number of WGMs in these bottles makes difficult the identification of all the WGMs in the spectrum. In addition, several notches centered at different wavelengths were fitted with a Lorentzian fit to estimate the *Q* factors. Those are defined by the ratio of the minimum wavelength and the linewidth of the fitted notch, *Q* = λ/∆λ. For this parabolic-profile bottle, WGMs possess Q values of around 10^6^.

**Figure 5:**
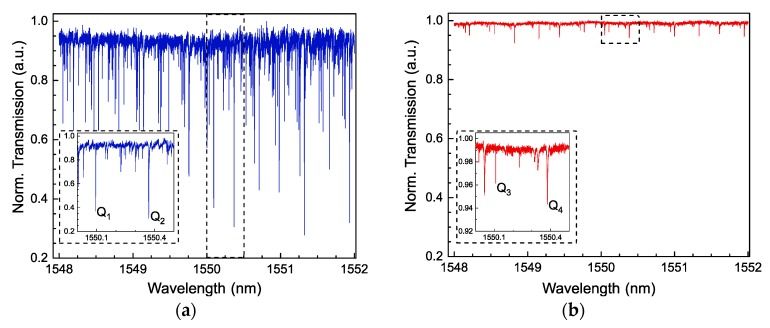
Transmission spectra of a bottle (D_c_ = 243 µm and L = 363 µm) interrogated with two taper diameters of (**a**) 5 µm and (**b**) 6.6 µm. Zoomed plots show some WGMs where quality (Q) factors were found to be Q_1_ ~ 1.91 × 10^6^, Q_2_ ~ 7.7 × 10^5^, Q_3_ ~ 7.38 × 10^6^, and Q_4_ ~ 4.4 × 10^5^. Step size was 0.1 pm.

Following the spectral characterization of the bottle structures, tapers with two different waist diameters were used to evanescently excite the corresponding WGMs. In both cases, the transmitted spectrum was measured by placing the taper in the center of the bottle (D_c_ = 243 µm and L = 363 µm). Figure 5a,b illustrates the resulting spectra when the nominal diameter of the taper was 5 and 6.6 µm. We have observed that the number of notches was drastically decreased due to the increasing of the taper diameter. However, the light coupled to the bottle resonator using a 6.6 µm diameter taper is only about 8%, as is shown in Figure 5b (zoomed windows). The reduction of WGMs in thicker fibers is originated by the phase conditions between the fiber mode and the WGMs. A similar phenomenon was reported in Reference [5] for cylinder resonators. In this context, smaller taper diameters excite more efficiently the WGMs in the bottle. About the Q factor; this was not seriously affected when we used thicker fibers. The Q values are given in the caption of Figure 5a,b.

**Figure 6:**
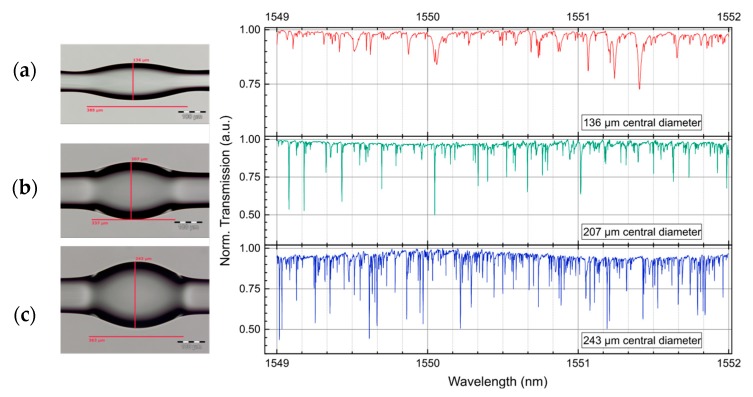
Transmission spectra obtained with three different bottle structures when a 5 µm diameter is located in the center. Bottle parameters are (**a**) D_c_ = 136 µm, L = 385 µm; (**b**) D_c_ = 207 µm, L = 337 µm, and (**c**) D_c_ = 243 µm, L = 363 µm. Step size was 0.1 pm.

WGM resonances have also been studied for three different types of bottle resonators. In this case, the taper diameter was kept constant at 5 µm. The bottle resonator in Figure 6a was formed from an 80 µm diameter single-mode fiber. The resulting geometry was D_c_ = 136 µm and L = 363 µm. Figure 6b,c shows bottle structures with D_c_ = 207 µm and L = 337 µm, and D_c_ = 243 µm and L = 363 µm. From the spectra, we can see that sine-profile bottles (>∆k) show a high number of narrow notches while parabolic-profile bottles (<∆k) exhibit a low number of broad notches. In other words, the *Q* factor decreases two orders of magnitude, from 10^6^ to 10^4^, when smaller parabolic-profile bottles are excited with thicker tapers. Note that the spectrum in Figure 6a is similar than the spectrum obtained with a cylinder resonator. This effect is tightly related to the modal coupling conditions between the bottle resonator and the taper.

### 3.2. Characterization of the Packaged Bottle

**Figure 7:**
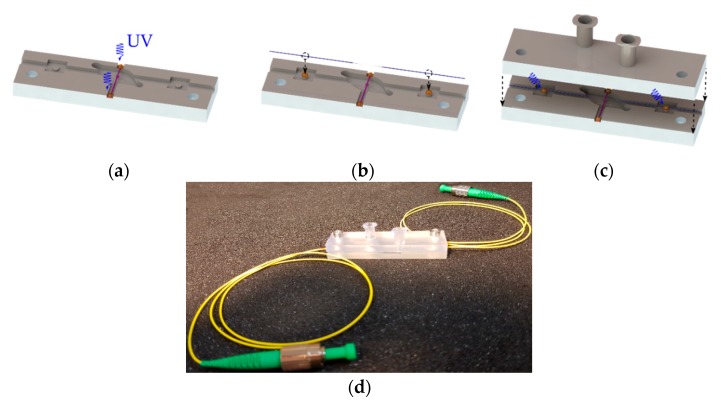
(**a**–**c**) Assembly process of a botte resonator; (**d**) first 3D-printed package of a taper-bottle system.

To exploit the thermal sensitivity of the WGMs, a bottle resonator was assembled into a robust plastic package. The bottle parameters were D_c_ = 207 µm and L = 300 µm. The assembly process was carried out as follows: As a first step, the bottle resonator was positioned inside the bottle channel of the plastic base and later fixed by dispensing a small drop of UV-curable epoxy adhesive, as shown in Figure 7a. The alignment of the bottle was assisted by a small stripe placed in the middle of the chamber. Afterwards, the 5 µm diameter taper was fabricated and aligned with respect to the glued bottle resonator as shown in Figure 7b. The alignment procedure was controlled using the optical setup described in Section 2.3. After observation of WGMs, the fiber was fixed by curing two small adhesive drops previously deposited on the two pillars, as shown in Figure 7c. During the curing step, a fine alignment was repeated to assure good optical coupling. Then, the cover unit was incorporated by a pair of screws and completely sealed with epoxy adhesive. The assembled package in Figure 7d was filled out with inert gas (nitrogen) in order to preserve a dust particle-free medium. Finally, the package was placed inside a box for temperature stabilization.

**Figure 8:**
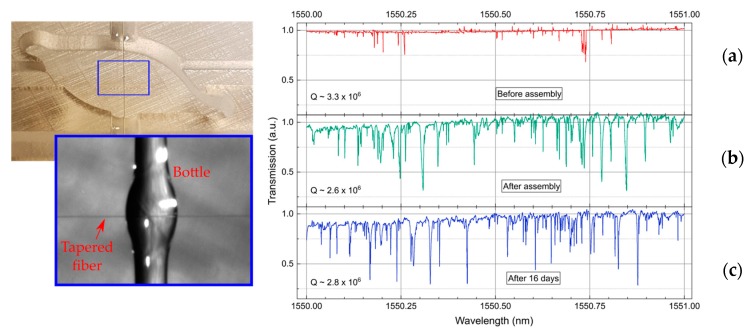
Optical characterization of the packaged bottle: (**a**) before assembly; (**b**) after assembly; and (**c**) after 16 days.

The WGM spectrum of the packaged bottle resonator was recorded before and after assembly, and additionally this was monitored during a period of 16 days. Before assembly, the spectrum in Figure 8a shows atypical notches where some of them present asymmetric line shapes. Such an effect can be related to the excitation of Fano resonances which can be observed with thicker (multimode) and non-adiabatic tapers [20,21]. As an upcoming study, the optical influence of the taper dimensions will be deeply investigated. Figure 8b displays the corresponding spectrum after assembly. Here, WGM resonances show normal symmetric line shapes. We believe that a shift along the taper could occur. In Figure 8c, the packaged bottle resonator was optically monitored over 16 days. By comparing the last two spectra, a slight variation at wavelength positions was observed. This can be related to a small misalignment of the taper with respect to the bottle, which could be caused by the shrinkage of the adhesive. The Q factors of WGMs have also been tracked from day 0 to day 16. We conclude that there is not a high degradation of the Q factor during the 16 days. However, an optimization of the package is being developed in order to assemble bottle resonators and tapers with better accuracy and mechanical stability. In regard to thermal stability, several approaches [22,23] based on negative thermo-optic coating materials could also be evaluated to compensate for temperature fluctuations without affecting the sensitivity of the bottle resonator. 

### 3.3. Temperature Measurements 

**Figure 9:**
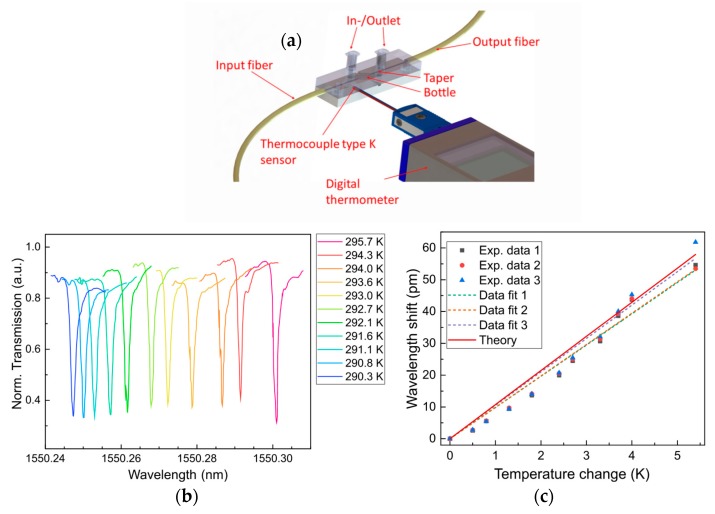
(**a**) Schematic of the packaged bottle resonator that includes an external digital thermometer; (**b**) spectral shift of a WGM resonance when temperature varies from 290.3 to 295.7 K; (**c**) wavelength shifts of three WGMs as a function of the temperature. Sensitivities are given in Table 3.

In order to demonstrate the sensing properties of the package from Figure 8, temperature changes were induced. These changes were generated and measured by the incorporation of a cooling/heating plate element and a calibrated thermocouple sensor. The sensor needle was in contact with the plastic package, as shown in Figure 9a, while the plate was located under the package. The temperature range of interest was from 290.3 to 295.7 K. As an example, Figure 9b shows the variation of a WGM as a function of the temperature. Two additional WGMs were also investigated. In Figure 9c, the wavelength shifts of these three WGM resonances initially centered around 1550.13, 1550.25, and 1550.58 nm were measured when the temperature increased. As expected, a linear red shift of 53 pm until 62 pm was observed. 

Table 3 summarizes the experimental parameters for three WGM resonances. Bottle resonators possess WGM resonances with different Q factors and, consequently, different sensitivities. A highest temperature sensitivity was found to be 10.5 pm/K, resulting in a limit of detection of 0.06 K. The experimental values were compared to the theoretical calculations realized for glass bottles in Reference [13] which depends mainly on the thermo-optic property of the glass material. We have found a theoretical sensitivity value of 10.73 pm/K. This value fits perfectly to the experimental sensitivity values of the packaged bottle reported in this work.

## 4. Conclusions

We conclude that glass bottle resonators assembled in a simple and robust 3D-printed package can be used to detect small temperature changes. A complete experimental characterization of bottle structures with parabolic and sine profile was successfully carried out. The highest *Q* factor value of 7.4 × 10^6^ was obtained with a sine-profile bottle structure (D_c_ = 243 µm and L = 363 µm). A theoretical analysis of a parabolic-profile bottle was also realized. The comparison of the theoretical and the experimental FSR values at two coupling positions shows good agreement. The optical influence of the taper diameter in WGMs has been assessed. However, a deep study is still needed to explain the possible observation of Fano resonances. We have demonstrated an assembly approach for fiber-coupled bottle resonators. The first package sensor was tested by temperature changes. The best temperature sensitivity was determined to be 10.5 pm/K. This value was perfectly matched to the theoretical sensitivity of 10.73 pm/K. These reported results make bottle resonators a promising and competitive sensor platform against other fiber-formed WGM structures.

Finally, improvements are still needed to bring optical microresonators close to practical prototypes. However, several strategies are being investigated to assemble fiber-based resonators in small cartridge packages to be exploited as highly-sensitive optical devices.

## Figures and Tables

**Table 1:** Calculated orders (*m*, *p*, *q*) of two typical whispering gallery modes (WGMs) around λ = 1550 nm for TE and TM polarization that correspond to the position of the taper with respect to the bottle resonator.

Position at z (µm)	(*m*, *p*, *q*) TE	(*m*, *p*, *q*) TM
0	(519, 1, 0)	(519, 1, 0)
110	(435, 1, 175)	(436, 1, 176)

**Table 2:** Experimental and theoretical FSRs for TE and TM WGMs calculated at z = 0 and z = 110 µm.

	Experimental FSR (nm)	Theoretical TE FSR (nm)	Theoretical TM FSR (nm)
FSR_azimuthal,1_	2.892	2.911	2.911
FSR_azimuthal,2_	2.902	2.921	2.921
FSR_azimuthal,3_	2.916	2.933	2.933
FSR_azimuthal,4_	2.892	2.912	2.912
FSR_azimuthal,5_	2.903	2.924	2.923
FSR_azimuthal,6_	2.916	2.935	2.935
FSR_axial,1_	1.3212	1.322	1.317
FSR_axial,2_	1.3248	1.324	1.321
FSR_axial,3_	1.3266	1.320	1.315

**Table 3:** Experimental parameters at 294 K for three different WGMs.

	Wavelength (nm) T = 294 K	Q T = 294 K	Sensitivity (pm/K)	Limit of Detection (K)
1	1550.1723	7.28 × 10^5^	9.8	0.22
2	1550.2867	1.25 × 10^6^	9.9	0.13
3	1550.6165	2.58 × 10^6^	10.5	0.06
